# Degradation of piroxicam and celecoxib from aqueous solution by high-energy electron beam as a Sustainable method

**DOI:** 10.1016/j.heliyon.2024.e39839

**Published:** 2024-10-24

**Authors:** Niloufar Borhani Yazdi, Mohammad Rezvani Ghalhari, Ali Parach, Mohammad Hassan Ehrampoush, Kamal Ghadiri, Mahdi Ghorbanian, Mohammad Hossein Zare Hassanabadi, Ehsan Abouee Mehrizi

**Affiliations:** a Department of Environmental Health Engineering, School of Public Health, Tehran University of Medical Sciences, Tehran, Iran; b Department of Medical Physics, Shahid Sadoughi University of Medical Sciences, Yazd, Iran; c Department of Environmental Health, School of Public Health, Shahid Sadoughi University of Medical Sciences, Yazd, Iran; d Environmental and Occupational Health Research Center, Shahroud University of Medical Sciences, Shahroud, Iran; e Vector-borne Diseases Research Center, School of Health, North Khorasan University of Medical Sciences, Bojnurd, Iran; f Student’s Scientific Research Center, Tehran University of Medical Sciences, Tehran, Iran

**Keywords:** Wastewater treatment, Nonsteroidal anti-inflammatory drugs, Advanced oxidation processes, High-energy electron beam irradiation

## Abstract

Non-steroidal anti-inflammatory drugs (NSAIDs) are one of the most commonly prescribed drugs that can reduce pain. This study aimed to measure the concentration of piroxicam and celecoxib in Iranian hospitals, as well as the effect of electron beam irradiation on the degradation of these pollutants in synthetic and real samples. The high-performance liquid chromatography (HPLC) was used to detect the residual analytes in the samples. The Response Surface Methodology (RSM) was used to design the experiment conditions that investigate the effect of electron beam irradiation on degradation of piroxicam and celecoxib from synthetic samples, and then according to the optimum condition, the experiments were carried out for real wastewater samples. The results of wastewater analysis shown that the mean concentration of PIRO and CELE were 6.32 ± 2.5 and 11.5 ± 3.2 μg/L, respectively. Also, the findings show that 98.98 % and 97.62 % of piroxicam and celecoxib was degraded, respectively, when the optimum conditions (pH = 4, electron beam irradiation = 8 kGy, and concentrations of 60 μg/L for piroxicam and 50 μg/L for celecoxib) were applied. Results show that the degradation rates of piroxicam and celecoxib in the real wastewater sample at optimum condition were 89.6 % and 84.25 %, respectively. So, electron beam irradiation is a long-lasting and promising method for removal emerging contaminants from wastewater, like non-steroidal anti-inflammatory drugs, that can't be removed by conventional wastewater treatment methods; so, it can be used in combination with conventional wastewater treatment methods.

## Highlights

• Electron beam irradiation used as a promising technique for Piroxicam and Celecoxib degradation.
• Piroxicam and Celecoxib were degraded 98.98 % and 9.62 %, respectively under optimized condition.
• Kinetic model of Piroxicam and Celecoxib degradation followed pseudo-first-order.

## Graphical abstract

**Figure:**
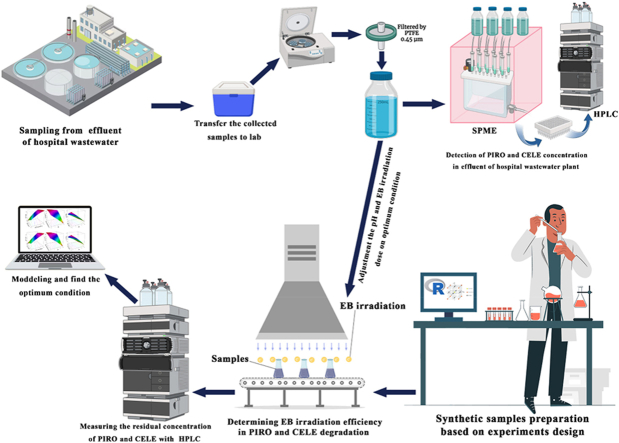

## 1 Introduction

Non-steroidal anti-inflammatory drugs (NSAIDs) are extensively used medications for treating a variety of conditions, including rheumatoid arthritis, inflammation, and fever [1,2]. Naproxen, Piroxicam (PIRO), Mefenamic Acid, Celecoxib (CELE), Indomethacin, Etoricoxib, Lornoxicam, Meloxicam, Diclofenac, Ibuprofen, Tenoxicam, and Valdecoxib are the primary NSAIDs that used in hospitals [3]. The residuals of them can discharge into wastewater [4]. The presence of these pollutants in water sources can potentially threat human health and the environment [5]. PIRO and CELE are water-soluble and have polar functional groups, so the conventional wastewater treatment processes are unable to remove the significant level of them [6]. During the COVID-19, in Iranian hospitals the PIRO and CELE were overused due to their antiviral effect; so, a large amount of them residues (from ng/L to mg/L) are entry into hospital wastewater [[7], [8], [9]].

PIRO is deposited in aquatic environments, promoting the growth of cyanobacterial populations via displacement of eukaryotic algae [10]. The presence of PIRO was identified in 20 % of the samples examined in the influent of five distinct wastewater treatment plants in Portugal. The average concentration of piroxicam was found to be 2.6 ng/L [11]. Continuous exposure to Piroxicam has been associated with alterations in behavior, damaging effects on reproduction, and physiological strain in fish species including *Danio rerio* (zebrafish) [12]. Exposure to Celecoxib can induce stunted development and reproductive failure in species such as Daphnia magna [13]. A further noteworthy issue is the possibility of bioaccumulation of these medications in aquatic organisms [14]. The consumption of contaminated food supplies by smaller species can lead to an increase in concentrations of Piroxicam and Celecoxib up the food chain, therefore possibly affecting larger predators and even humans who consume fish and shellfish [15]. The persistence and stability of PIRO and CELE in water environments have caused major ecological concerns; so, these pollutants should be removed from aqueous solutions even in the trace concentration [16].

Conventional methods of wastewater treatment alone are disable to effectively remove these pollutants, so the use of new methods can increase the removal percentage [17]. advanced oxidation process (AOPs), sonication and electrolysis techniques are the novel methods that can combine with conventional methods and can enhance removal rate [[17], [18], [19]]. Advance oxidation processes (AOP) use the extremely reactive and non-selective hydroxyl radical (^•^OH) to oxidize a variety of compounds; thus, wastewater treatment with AOPs reduces organic pollutants and improves industrial effluent biodegradability [20]. The electron beam (EB) irradiation is one of the AOPs which can increase the removal rate of NSAIDs such as PIRO and CELE [21,22].

EB irradiation also disinfects wastewater and sewage sludge, making eco-friendly fertilizers [23]. In recent years, Rhodotron accelerators generated reactive species with high-energy EB which can degrade the emerging contaminants. Wide use of NSAIDs like PIRO and CELE created environmental concerns [24,25]. Although PIRO and CELE are widely used as NSAIDs in Iranian hospitals, there is insufficient evidence to determine their residual levels in hospital wastewater; also, there is no research evidence that shows the effect of EB irradiation in the degradation of PIRO and CELE from aqueous solutions. The present study aimed to: 1) analyze hospital wastewater and measure the concentration of PIRO and CELE, which are classified as highly-rated NSAIDs in Iranian hospitals; 2) investigate the impact of the EB radiation process on the degradation of PIRO and CELE in synthetic samples and determine the optimal conditions for this process; 3) examine the degradation of PIRO and CELE in real hospital wastewater samples under optimal conditions.

## 2 Materials and methods

### 2.1 Materials

The analytical standards for NSAIDs, specifically PIRO (C_15_H_13_N_3_O_4_S) (≥98 %) and CELE (C_17_H_14_F_3_N_3_O_2_S) (≥98 %), were obtained from Sigma-Aldrich (St. Louis, USA). Additionally, the extracting solvent 1-dodecanol (≥98 %) was also given by Sigma-Aldrich. The methanol (MET) and acetonitrile (CH₃CN) used in this study were of high-performance liquid chromatography (HPLC) grade and were obtained from Merck (Darmstadt, Germany). The acquisition of ultrapure water was accomplished by the utilization of a Milli-Q Ultra purification system with a conductivity of 18.2 MΩ cm^−1^ (Milli-Q Millipore, Bedford, USA). The pH of the samples was adjusted by a pH meter (ORION™ Star A211). Stock solutions of each analytical standard, with a concentration of 100 mg L^−1^, were produced using methanol as the solvent. The operative solutions were concocted by diluting the original solution in ultrapure water to get the targeted concentrations. The TT200 Rhodotron accelerator irradiation is one of the equipment that can produce high-energy EB and can help to degrade the PIRO and CELE under controlled conditions.

### 2.2 Solid phase microextraction (SPME)

The SPME method was conducted by injecting a mixture consisting of 150 μL of acetonitrile and 30 μL of 1-dodecanol as extracting solvent. This mixture was promptly transferred to a conical tube containing 5 mL of aqueous sample that had been acidified to pH 2 with 85 % H3PO4 (v/v) and NaCl 2.5 % (m/v). The tube was agitated using a vortex mixer (Vortex MX-S, Scilogex, Bedfordshire, UK) for 20 s, followed by centrifugation at 5000 rpm for 4 min using a Q222T centrifuge (Quimis, Diadema, Brazil). The resulting mixture was then placed in an ice bath for 12 min to solidify the organic phase. The solid extract was extracted, transferred to a 2 mL vial, and left to liquefy at room temperature. Subsequently, it underwent analysis using HPLC equipped with a diode array detector (DAD). Before conducting HPLC analysis, all samples were subjected to filtration using a 0.45 μm nylon filter membrane from Millipore (Darmstadt, Germany) [26].

### 2.3 Chromatographic analysis

PIRO and CELE were identified using the HPLC (Shimadzu, Kyoto, Japan) model SPD-M30A coupled to Diode-Array Ultraviolet Detector (HPLC-DAD-UV). The substances being analyzed were separated using a reversed-phase column called Phenomenex Luna C18, which had dimensions of 250 × 4.6 mm and included 5 μm particles [27]. The mobile phase used for separation consisted of a mixture of acetonitrile and water, with the water being acidified to a pH of 2 using 85 % phosphoric acid (H_3_PO_4_) [28]. The ratio of acetonitrile to water in the mobile phase was 60:40 (v/v), and the flow rate of the mobile phase was 1.2 mL. The absorbance of PIRO and CELE were measured at a wavelength of 350 nm using a Shimadzu HPLC with a DAD detector. The flow rate was maintained isocratically at 0.18 mL/min using a mixture of 75 % water with 0.1 % phosphoric acid and 25 % acetonitrile [29].

The calibration curves provide information on the limit of detection (LOD) and limit of quantification (LOQ). Also, according to Eq. (1) and Eq. (2) the LOD and LOQ were achieved [30,30].

Eq.1 $$LOD=3\times \frac{S_{1}}{S_{0}}$$

Eq.2 $$LOQ=10\times \frac{S_{1}}{S_{0}}$$

Where the S_0_ presents the slope of calibration curve and S_1_ describes the standard deviation of the regression equation.

### 2.4 EB irradiation setup

**Fig. 1:**
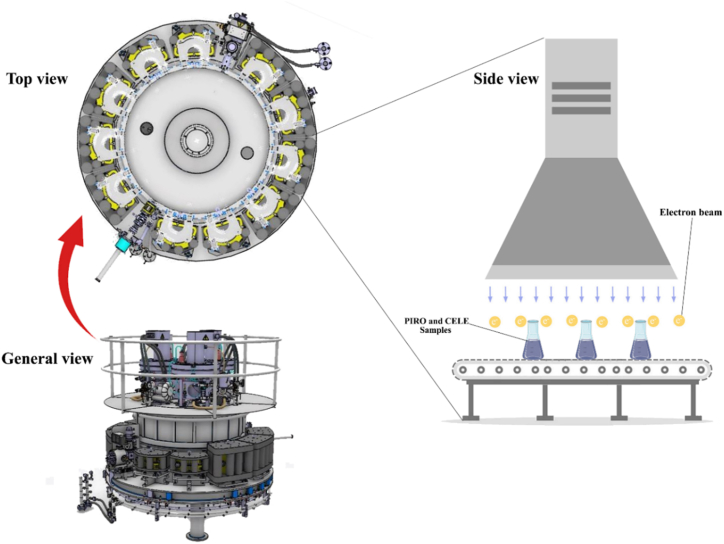
TT200 Rhodotron electron beam accelerator schematic.

The experimental setup utilized the TT200 Rhodotron EB accelerator, which is widely recognized as one of the most sophisticated and modern accelerators worldwide (see Fig. 1). The TT200 Rhodotron EB accelerator operates at an energy level of 10 MeV, with a beam current of 10 mA and a beam power of 100 kW. The absorbed dose was calibrated utilizing a GEX B3 dosimeter, and in order to safeguard the samples, borosilicate containers (Pyrex) were encased with plastic wrap during the process of irradiation. The properties of TT200 Rhodotron accelerator presented in Table 1.

**Table 1:** Properties of the TT200 Rhodotron accelerator equipment.

Properties	Amounts
Beam Power at 5 Megaelectron Volts	35 kW
Beam Power at 10 Megaelectron Volts	75 kW
Energy Scatter at 10 Megaelectron Volts	±300 keV
Sweep Range	30–100 cm
Overall, Power Consumption	≤300 kW
Radio Frequency (RF)	107.5 MHz
RF output power	200 kW
Electron gun medium current	0–10 mA
precision	±50 μamps

### 2.5 Sample collection

Three samples of wastewater were collected by grab sampling of mixed wastewater taken at three different locations from the equalization tank at the Yazd-based Shah-Vali Hospital's effluent treatment plant (ETP) then the samples were placed in sample bottles of amber hue and promptly transported to the laboratory, where they were afterwards maintained at a temperature of 4 °C until the analysis was conducted and subsequently filtered by PTFE 0.45 μm to eliminate any particle matter. Finally, we adjusted the pH of the collected samples on pH = 5 by HCl 1N (PIRO_pKa_ and CELE_pka_ were 5 and 5.4, respectively) for the SPME process [31].

### 2.6 Experimental design, optimization, and statistical analysis

The central composite design (CCD) as a popular subset of response surface methodology (RSM) has been used to determine the experimental situation of PIRO and CELE degradation via EB irradiation using R software, version 3.6.0 (Missouri, USA). In the present study, the pH range was considered 2–10, the range of EB irradiation dose was 5–20 kGy, and the range of both NSAIDs concentrations was considered 50–100 μg/L, according to the literature review (see Table 2). Due to the high degradation rate of EB irradiation, the degradation process was carried out in a fraction of a second, and changing the time did not cause a significant change in the efficiency of degradation, so it was not possible to consider time as a variable. The designed experiment situation and responses of each run are presented in Table 4. The experiments were carried out under controlled conditions at a constant temperature of 20 ± 0.5 °C in a batch reactor. The statistical significance of the fitted quadratic models, relevant factors, and their interactions on the response variable was also assessed using analysis of variance (ANOVA) tests with a significance level of P < 0.05. Additionally, the coefficient of determination (R^2^) and a linear regression model were employed for further investigation. Also, the user-friendly tool "Microsoft Excel's Solver" can be utilized to determine the optimal value for each parameter.

**Table 2:** The main values of parameters that effect on the degradation of PIRO and CELE via EB irradiation considered for CCD.

Alpha	pH	EB dose (kGy)	PIRO (μg/L)	CELE (μg/L)
+2	10	20	100	100
+1	8	17	90	90
0	6	12	75	75
−1	3	8	60	60
−2	2	5	50	50

**Table 3:** The physicochemical characteristics of wastewater treatment plant of Yazd-based Shah-Vali hospital (n = 3).

Parameter	Symbol	Unit	Mean	±SE
Potential of hydrogen	pH	unitless	7.24	0.357
Suspended solid	SS	mg/L	560.5	49.88
Chemical oxygen demand	COD	mg/L	790	87.23
Biological oxygen demand	BOD_5_	mg/L	442.5	35.39
Total nitrogen	TN	mg/L	156.9	50.51
Total phosphorus	TP	mg/L	14.275	8.29
Piroxicam concentration	PIRO	μg/L	6.32	1.44
Celecoxib concentration	CELE	μg/L	11.5	1.84

**Table 4:** Independent degradation variables and result in the degradation percentage of PIRO and CELE via EB irradiation.

Run	pH	EB irradiation dose (kGy)	Initial concentration (μg/L)	PIRO degradation (%)	CELE degradation (%)
1	8	8	90	68.36	56.99
2	8	8	60	70.18	58.77
3	6	12	75	91.19	79.34
4	8	17	60	86.19	74.44
5	3	17	60	98.21	87.24
6	3	8	90	87.10	75.34
7	6	12	75	83.17	71.48
8	3	17	90	95.20	83.28
9	6	12	75	78.16	66.58
10	3	8	60	93.80	81.91
11	8	17	90	89.19	77.39
12	6	5	75	65.10	53.81
13	6	12	75	88.18	76.40
14	6	12	100	72.15	60.70
15	2	12	75	97.19	87.24
16	6	12	75	85.19	73.46
17	6	20	75	99.21	90.23
18	6	12	50	94.20	82.31
19	6	12	75	79.17	67.57
20	10	12	75	69.16	53.78

The removal efficiency of PIRO and CELE was subsequently calculated using **Eq. (3)** [32].

Eq. 3 $$Removal\;efficiency\;\left(\%\right)=\left[\frac{\left(C_{0}-C_{t}\right)}{C_{0}}\right]\times 100$$

Where C_0_ is the initial concentration of the analytes (PIRO and CELE), and C_t_ is the concentration of the PIRO and CELE at time t.

### 2.7 Simultaneous Quantitative analysis of PIRO and CELE in complex matrices

The PIRO and CELE have overlapping spectral signals within different linear ranges. So, a novel approach was employed to enhance signal measurement accuracy for the simultaneous analysis of PIRO and CELE concentrations. Extensive optimization of variables was conducted to ensure optimal performance of the method. The proposed approach allowed for simultaneous measurements of these compounds, even in the presence of interfering agents that exhibit spectral overlap. To determine the concentrations of both compounds, the method utilized the concept of pure analyte signal and employed chemometrics techniques.

The standard addition method, specifically the net analyte signal standard addition method (NASSAM), was implemented in this study. This approach involves adding a known quantity of analyte standard solution to a portion of the sample and measuring the responses before and after the addition to determine the analyte concentration in complex matrices solutions. Multiple additions were performed alternately in different parts of the sample to enhance accuracy. It is important to note that the standard addition method was employed in situations where replicating the sample matrix was challenging or impossible. This innovative method demonstrated high sensitivity, selectivity, and cost-efficiency in simultaneously measuring PIRO and CELE, effectively overcoming challenges associated with spectral overlap and interferences.

In this experimental approach, a fixed volume (V_unk_) of the unknown solution was introduced into four separate volumetric flasks (V_flask_). Successively, incremental volumes of a control solution (V_std_) were added to each flask. The flasks were then filled with solvent up to the mark and thoroughly mixed. The selection of the concentration and volume of the added control solution aimed to achieve an approximate 30 % increase in the unknown concentration with each successive flask. The actual concentration of the analyte in each flask was determined using Eq. (4):

Eq. 4 $$C_{\text{unk}}=\frac{\left(C_{0}\times V_{\text{unk}}+C_{\text{std}}\times V_{\text{std}}\right)}{V_{\text{flask}}}$$

The relationship between the instrumental response, denoted as R, and the concentration of the analyte, represented as K, can be expressed through a mathematical function (See Eq. 5, Eq.6):

Eq. 5 $$R=K\;\left(\frac{C_{0}\times V_{\text{unk}}}{V_{\text{flask}}}\right)+K\;\left(\frac{C_{\text{std}}\times V_{\text{std}}}{V_{\text{flask}}}\right)$$

To simplify the notation, CSA (Control Solution Addition) was defined as $\frac{C_{std}\times V_{std}}{V_{flask}}$. So the R calculated according to Eq. (6):

Eq.6 $$R=K\;\left(\frac{C_{0}\times V_{unk}}{V_{flask}}\right)+K\;\left(CSA\right)$$

To determine the unknown response value (CSA) and subsequently calculate the analyte concentration, a linear regression model was employed using Eq (7). By plotting the responses obtained from the series of standard additives, the extrapolation of y = 0 enabled the estimation of CSA.

Eq. 7 y=b+mx

## 3 Results and discussion

### 3.1 NSAIDs concentration and physicochemical characteristics

Table 3 presented the physicochemical properties of collected wastewater. Also, the concentration of PIRO and CELE in the effluent of wastewater treatment plant of Yazd-based Shah-Vali hospital analyzed which the mean concentration of PIRO and CELE were 6.32 ± 2.5 and 11.5 ± 3.2 μg/L, respectively. Molnarova et al. (2023) analyzed drinking water samples which they results shown that the mean concentration of PIRO in samples were 9.93 ng/L [33]. Also, Patel et al. (2019) measure the concentration of PIRO in hospital effluent which the results of their analysis shown that the PIRO concentration is 51 ng/L, which is very lower than the concentration of PIRO in present study that analysis the PIRO concentration in the effluent of a hospital wastewater [34]. The results of the Triñanes et al. (2015) study's shown that the mean concentration of CELE was 49.4 ± 2.5 [35]. In present study, the LOD and LOQ for PIRO were 0.05 μg/L,and 0.14 μg/L, respectively. Also, the LOD and LOQ for detection of CELE were 0.09 μg/L, and 0.21 μg/L respectively. The determination coefficient (r^2^) for PIRO and CELE were 0.9969, and 0.9978, respectively.

### 3.2 Effect of EB irradiation on the removal of NSAIDs

The effect of EB irradiation on the removal of NSAIDs from aqueous solutions was investigated by focusing on assessing the efficacy of the Rhodotron TT200 accelerator. Table 4 present the results of PIRO and CELE degradation by EB irradiation under designed experiments. Results shown that the run number 17 has the most degradation %, which were 99.21 % and 90.23 % for PIRO and CELE, respectively. The most degradation % have occurred when the pH, EB irradiation and analytes concentration were 6, 20 kGy and 75 μg/L, respectively. The most degradation occur at a situation that is not economic because required very EB irradiation which can use a huge energy that is not cost-benefit, so the optimization is necessary to find the best situation which can have the most degradation % under economic and engineering condition.

### 3.3 Radiolytic degradation of PIRO and CELE for environmental remediation

The process of utilizing EB radiation for the degradation of emerging contaminants in water entails the generation of primary products via the phenomenon of water radiolysis. The aforementioned products encompass hydrogen atoms (H), hydrated electrons (eaq^−^), hydroxyl radicals (^**·**^OH), and comparatively less reactive species like H_3_O^+^ (**Eq. (8)**) [36]. The formation of these species occurs due to the interaction between an electron beam and water molecules, and they play a vital role in the degradation process that takes place during electron beam irradiation. Under specific conditions during EB radiation in an aqueous solution, the radicals interact and lead to several outcomes: (i) In the presence of dissolved oxygen, the H· and ea_q_^−^ radicals convert to HO_2_^·^ and O_2_^·−^, as represented by **Eqs. 9** and **10**; (ii) At higher radiation doses, the reaction between ^·^OH and ea_q_^−^ can be described by **Eq. (11)**; (iii) In acidic conditions, H^+^ reacts readily with ea_q_^−^ to produce H^·^, as shown in **Eq. (12)**; (iv) The equilibrium between HO_2_^·^ and its conjugate base O_2_^·−^ is pH-dependent, as illustrated in **Eq. (13)**; (v) Additionally, gamma radiation may lead to various other reactions, as demonstrated by **Eqs. (14)–(16**) [37].

**Table:** 

*H*_*2*_*O→e*^*−*^_*aq*_*(2.6) + OH + H· (0.55) + ·OH (2.7) + H*_*2*_*(0.45) + H*_*2*_*O*_*2*_*(0.71) + H*_*3*_*O*^*+*^*(2.6)*	*Eq. (8)* [38]
*H· + O*_*2*_*= HO*_*2*_*· k = 2.1 × 10*^*10*^*mol*^*−*^*^1^ s*^*−*^*^1^*	*Eq. (9)* [39]
*e*^*−*^_*aq*_*+ O*_*2*_*= O·*_*2*_^*−*^*k = 1.9 × 10*^*10*^*mol*^*−*^*^1^ s*^*−*^*^1^*	*Eq. (10)* [40]
*e*^*−*^_*aq*_*+ ·OH = OH*^*−*^*k = 3.0 × 10*^*10*^*mol*^*−*^*^1^ s*^*−*^*^1^*	*Eq. (11)* [41]
*e*^*−*^_*aq*_*+ H*^*+*^*= H· k = 2.3 × 10*^*10*^*mol*^*−*^*^1^ s*^*−*^*^1^*	*Eq. (12)* [41]
*HO·*_*2*_*= O·*_*2*_^*−*^*+ H*^*+*^*k = 8 × 10*^*5*^*mol*^*−*^*^1^ s*^*−*^*^1^*	*Eq. (13)* [40]
*HO·*_*2*_*+ O·*_*2*_^*−*^*= H*_*2*_*O*_*2*_*+ O*_*2*_*(pH < 7) k = 9.7 × 10*^*7*^*mol*^*−*^*^1^ s*^*−*^*^1^*	*Eq. (14)* [42]
*HO·*_*2*_*+ HO·*_*2*_*= H*_*2*_*O*_*2*_*+ O2 k = 8.3 × 10*^*5*^*mol*^*−*^*^1^ s*^*−*^*^1^*	*Eq. (15)* [42]
*H· + OH− = e*^*−*^_*aq*_*+ H*_*2*_*O k = 2.2 × 10*^*7*^*mol*^*−*^*^1^ s*^*−*^*^1^*	*Eq. (16)* [42]

EP is an advanced variant of the peroxone process, in which H₂O₂ is generated at the cathode and reacts with ozone to produce ^**·**^OH [43,44]. The present methodology effectively tackles the challenges inherent in direct ozonation, including the limited half-life of ozone in water and its selective oxidation of certain contaminants. The EB method generates a greater diversity of reactive compounds by the use of electron beam radiation, thereby enhancing its efficacy in the degradation of a broader spectrum of contaminants. Reacts ozone with in-situ produced hydrogen peroxide to produce hydroxyl radicals. The interaction between H₃O₃ and O₃ enhances the effectiveness degradation of pollutant [44]. Generating ^**·**^OH and other reactive species directly by electron beam irradiation provides a wider oxidation route without the need for external chemical reagents [45].

### 3.4 Influence of variables on PIRO degradation rate

The ANOVA analysis of PIRO degradation via EB irradiation process presented in Table 5. The results showed that pH increasing has a negative effect on PIRO degradation, so decreasing the pH level can help to increase degradation. It should be note that the pH decreasing can bring economic and engineering limitations, so optimum pH values should be selected considering these criteria. Also, it is an undeniable fact that increasing the dose of EB irradiation can increase the rate of degradation, but the optimum amount must be obtained in order to economically justify the use of radiation to removal various pollutants from wastewater. By using the optimum values and taking into account the synergistic effect of other parameters, it is possible to observe the highest PIRO degradation % with the lowest radiation cost. **Eq. (17)** can help to find the optimum condition using Solver ribbon. The results of optimization shown that the optimum condition of pH, EB irradiation, and PIRO concentration were 4, 8 kGy, 60 μg/L, respectively. In the optimum condition, about 98.98 % of PIRO can degrade, which is an acceptable degradation %; because the detected level of PIRO in the wastewater is lower than the optimum concentration, and since the rate of degradation increases with the decrease in analytes concentration, by observing other optimum conditions for concentrations less than 60 μg/L, it is possible to reach efficiencies close to 100 %. In present study, the degradation of PIRO from wastewater fitted to the quadratic model (R^2^ = 0.85, R^2^_adj._ = 0.77, p-value = <0.001 and the lack of fit = 0.125). After determining the optimum conditions (pH = 4, EB irradiation = 8 kGy, PIRO concentration = 60 μg/L), we investigated the PIRO degradation efficiency under optimum conditions to confirm the process's efficiency. The results indicated that 96.08 % of PIRO degraded under optimum conditions.

**Table 5:** ANOVA test for CCD modelling for PIRO degradation via EB irradiation and results of process optimization.

Piroxicam	Estimate	standard error	t value	p value	Sig.
Intercept	85.66785	1.11336	76.9454	<0.001	∗∗∗
pH	−12.84509	1.95224	−6.5797	<0.001	∗∗∗
EB	13.75015	1.95726	7.0252	<0.001	∗∗∗
PIRO	−5.28996	1.96201	−2.6962	0.014	∗
pH∗EB	8.93336	3.99072	2.2385	0.037	∗
pH∗PIRO	2.41258	4.00001	0.6031	0.55	
EB∗PIRO	3.66201	4.19011	0.874	0.39	
pH∗pH	−0.98178	2.80856	−0.3496	0.73	
EB∗EB	−2.82249	2.92852	−0.9638	0.35	
PIRO∗PIRO	−0.86875	2.90545	−0.299	0.77	
Multiple R-squared	0.8502				
Adjusted R-squared	0.7792				
p-Value	<0.001				∗∗∗

**Optimized values**					
**Parameter**	**Value**	**Eq. 17**			

pH	4	Y = 85.66+(-12.84∗pH) + (13.75∗EB) + (−5.28∗PIRO) + (8.93∗pH∗EB)			
EB irradiation dose (kGy)	8				
concentration (μg/L)	60				
R (%)	98.98				
**Analysis of Variance**					
	**Df**	**Sum sq**	**mean sq**	**f value**	**p value**

First order (pH, EB, PIRO)	3	1836.59	612.2	33.46	8.73E-08
Two-way interaction (pH, EB, PIRO)	3	111.94	37.31	2.04	0.1424
Pure quadratic (pH, EB, PIRO)	3	23.98	7.99	0.44	0.7291
Residuals	19	347.58	18.29		
Lack of fit	5	149.16	29.83	2.10	0.1253
pure error	14	198.42	14.17		

Signif. codes: <0.001 ‘∗∗∗’ 0.001 ‘∗∗’ 0.01 ‘∗’ 0.05 ‘.’ 0.1 ‘’ 1.

**Fig. 2:**
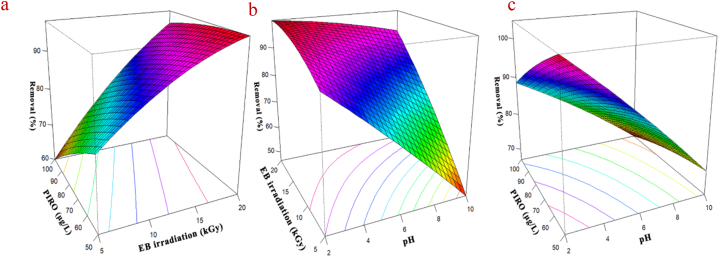
Three-dimensional (3D) response surface plots of the effect of EB irradiation (a), pH (b) and analyte concentration (c) on the PIRO degradation process.

To investigate the impact of initial drug concentration on the removal efficiency of PIRO during the EB irradiation process, a series of experiments was conducted at a temperature of 50 °C. The three-dimensional (3D) response surface plots of the effect of EB irradiation (Fig. 2a), pH (Fig. 2b) and PIRO concentration (Fig. 2c) on the degradation of PIRO via EB irradiation presented in Fig. 2. According to the results, with increasing the initial concentration of PIRO from 50 to 100 μg/L, the degradation efficiency decreased from 94.2 % to 72.15 %.

### 3.5 Influence of variables on CELE degradation rate

Table 6 shows the ANOVA analysis of the CELE degradation process using EB irradiation. The results show that increasing the pH level has a negative effect on CELE degradation, so decreasing the pH level can help to increase the degradation. It should be noted, however, that decreasing the pH level can bring economic and engineering limitations, so optimum pH values should be selected taking these criteria into account. Furthermore, while raising the dosage of EB irradiation might enhance the rate of degradation, the optimal quantity must be determined in order to economically justify the use of radiation to remove different contaminants from wastewater. It is feasible to observe the maximum CELE degradation with the lowest irradiation cost by applying the optimal condition and taking into consideration the synergistic impact of other factors. **Eq. (18)** can assist in determining the best condition that is economical and optimal using Solver ribbon. The optimization findings showed that the optimal pH, EB irradiation, and CELE concentration were 4, 8, and 50 μg/L, respectively; which the CELE degradation was 97.62 %. The detected level of CELE in the wastewater is lower than the optimum concentration, and because the rate of degradation increases with the decrease in analytes concentration, it is possible to achieve efficiencies close to 100 % by observing other optimum conditions for concentrations less than 50 μg/L. The degradation of CELE from wastewater in this investigation was fitted to a quadratic model (R^2^ = 0.86, R^2^_adj._ = 0.79, p-value <0.001, and lack of fit = 0.077). After determining the optimum conditions (pH = 4, EB irradiation = 8 kGy, CELE concentration = 50 μg/L), we investigated the CELE degradation efficiency under optimum conditions to confirm the process's efficiency. The results indicated that 94.71 % of CELE degraded under optimum conditions.

**Table 6:** ANOVA test for CCD modelling for CELE degradation via EB irradiation and results of process optimization.

Celecoxib	Estimate	standard error	t value	p value	Sig.
Intercept	74.02658	1.13847	65.0229	2.20E-16	∗∗∗
pH	−14.095	1.99627	−7.0607	1.02E-06	∗∗∗
EB	14.20495	2.0014	7.0975	9.45E-07	∗∗∗
CELE	−5.29064	2.00626	−2.6371	0.016	∗
pH∗EB	8.74725	4.08072	2.1436	0.045	∗
pH∗CELE	2.72382	4.09023	0.6659	0.51	
EB∗CELE	3.23013	4.28461	0.7539	0.46	
pH∗pH	−2.4128	2.8719	−0.8401	0.41	
EB∗EB	−1.37446	2.99456	−0.459	0.65	
CELE∗CELE	−0.93458	2.97098	−0.3146	0.76	
Multiple R^2^	0.86				
Adjusted R^2^	0.79				
p-Value	2.77E-06				

**Optimized values**					
**Parameter**	**Value**	**Eq. 18**			

pH	4	Y = 74.02+(-14.09∗pH) + (14.2∗EB) + (−5.29∗CELE) + (8.74∗pH∗EB)			
EB irradiation dose (kGy)	8				
concentration (μg/L)	50				
R (%)	97.62				
**Analysis of Variance**					
	Df	Sum sq	mean sq	f value	p value

First order (pH, EB, CELE)	3	2045.19	681.73	35.64	5.29E-08
Two-way interaction (pH, EB, CELE)	3	107.64	35.88	1.88	0.16796
Pure quadratic (pH, EB, CELE)	3	23.05	7.68	0.40	0.75344
Residuals	19	363.44	19.13		
Lack of fit	5	172.78	34.56	2.54	0.07779
pure error	14	190.66	13.62		

**Fig. 3:**
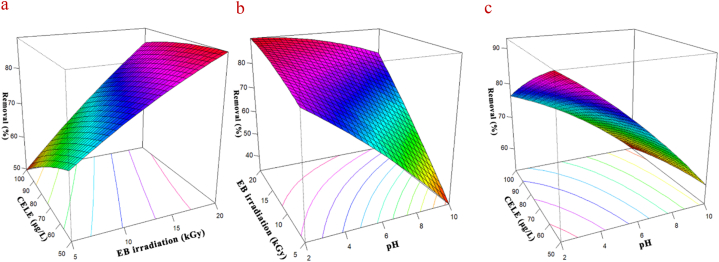
Three-dimensional (3D) response surface plots of the effect of EB irradiation (a), pH (b) and analyte concentration (c) on the CELE degradation process.

A series of studies was done at a temperature of 50 °C to examine the influence of the initial concentration of NSAIDs on the efficacy of CELE degradation during the process of EB irradiation. The three-dimensional (3D) response surface plots of the effect of EB irradiation (Fig. 3a), pH (Fig. 3b) and CELE concentration (Fig. 3c) on the degradation of CELE via EB irradiation presented in Fig. 3. According to the results, an increase in the starting concentration of CELE from 50 to 100 μg/L resulted in a drop in degradation efficiency from 82.31 % to 60.7 %. This decline may be attributed to the production of additional by-products during the degradation process.

### 3.6 Efficacy of EB radiation for degradation of NSAIDs in real samples

**Fig. 4:**
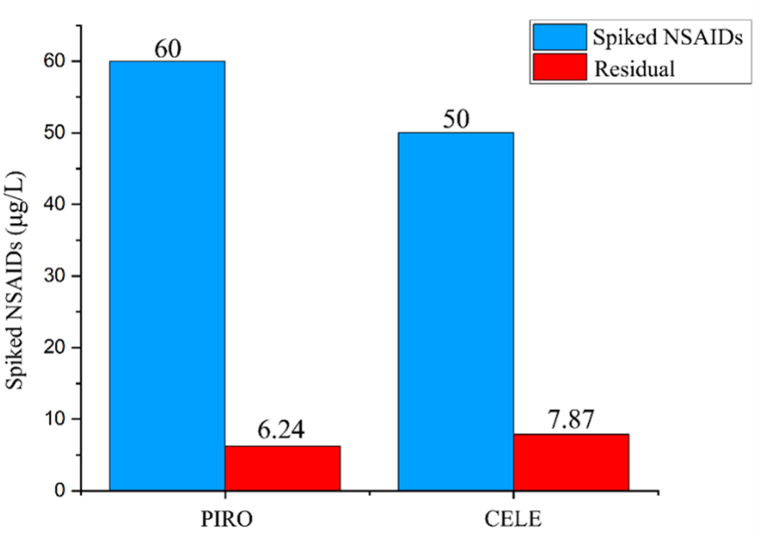
The effect of EB irradiation on PIRO and CELE degradation that spiked in real wastewater.

Finally, the real hospital wastewater was collected and the effect of EB irradiation on the PIRO and CELE degradation was investigated. As presented in Table 1 the mean (SD) concentration of PIRO and CELE were 6.2 ± 2.5 and 11.5 ± 3.2 μg/L. The concentration of the NSAIDs in the real samples are variable, so a specific concentration of PIRO and CELE should be spiked into the wastewater sample in which the concentration of PIRO and CELE cannot be detected; this process will reduce the analysis error. After sampling from wastewater free of PIRO and CELE, then 60 μg/L and 50 μg/L of PIRO and CELE were spiked to them, respectively. Presence of natural organic matters (NOMs) can reduce the degradation rate in the real situation [46]. Low photocatalytic efficiency is often the consequence of NOMs competing with PIRO and CELE for the consumption of reactive oxygen species (ROS) such ^•^OH or O_2_^−•^ radicals in actual wastewater [47,48]. As presented in Fig. 4, the PIRO degradation rate by EB irradiation using TT200 Rhodotron electron beam accelerator was 89.6 % (residual concentration = 6.24 μg/L) under optimum condition (concentration of PIRO = 60 μg/L, pH = 4, and EB irradiation dose = 8 kGy). Also, the degradation rate of spiked CELE into real wastewater under optimum condition (concentration of CELE = 50 μg/L, pH = 4, and EB irradiation dose = 8 kGy) was 84.25 % (residual concentration = 7.87 μg/L). These results highlight the potential of the EB radiation process for effectively removing CELE from wastewater samples, although the removal efficiency for PIRO was comparatively lower. It is essential to consider that the actual removal efficiency may vary depending on the specific characteristics of the wastewater samples and the optimization of process parameters. The degradation rate of ibuprofen by rGO@MnO_2_ which Hasan et al. (2023) carried out was 61.79 % in natural lake water and 93.49 % in deionized water [46], like the present study, the presence of NOMs can be one of the reasons for reducing the degradation efficiency.

### 3.7 Challenges and recommendations for future works

EB irradiation is promising technology for NSAIDs degradation in wastewater. For successful implementation, scaling up EB irradiation must overcome numerous challenges and it is difficult [49,50]. NSAIDs degradation requires optimizing all parameters that effect on degradation rate such as electron beam uniformity, wastewater electron penetration depth, and reactor architecture which is difficult for a large-scale plant; EB technology needs major changes to current infrastructure in order to be scaled up. When making electron beam accelerators, the size of the beam, how deep it goes, and how much energy they produce must all be taken into account for large-scale operations. It's possible that current systems can't easily be changed to handle bigger amounts of wastewater, so custom engineering solutions are needed [51]. EB facilities require large initial investments for electron accelerators and wastewater treatment infrastructure [52]. Depending on the size and needs of the company, an average EB system can cost anywhere from $1 million to $4 million. The cost of the equipment, its installation, and its connection to the current infrastructure are all part of this investment. Operational and maintenance costs can be substantial, making this technique less economically viable than standard treatment methods. EB systems usually have costs for things like power, repairs, and labor. One of the good things about e-beam technology is that it uses less energy than heat processes. Studies show that e-beam systems might only need 20–30 % of the energy that regular heat treatments do. This could mean lower long-term costs for running the system. Installation of EB accelerators in wastewater treatment plants can cost several hundred thousand to millions of dollars, depending on scale and technology [53,54]. Electron beam accelerators need a lot of electricity; therefore, electricity costs can be important. However, technology is making systems more energy-efficient, which may lower operational expenses [55,56]. EB irradiation should be compared to activated sludge, advanced oxidation, and membrane filtering for efficiency and cost [21,22]. The mentioned traditional methods are cheaper than EB, but they use more chemicals and may not remove contaminants as well [57]. EB irradiation can damage to personnel; therefore, it is necessary to observe safety measures in order to reduce these injuries [58].

## 4 Conclusion

The present study analyzed the concentrations of PIRO and CELE using the HPLC technique, and investigated the effects of EB irradiation under various conditions on their degradation. The environmental monitoring of Shah-Vali Hospital's wastewater in Yazd shows that the mean ± SD concentration of PIRO in the wastewater is 6.2 ± 2.5 μg/L, while the mean ± SD concentration of CELE is 11.5 ± 3.2 μg/L. The findings show that 98.98 % and 97.62 % of piroxicam and celecoxib was degraded, respectively, when the optimum conditions (pH = 4, electron beam irradiation = 8 kGy, and concentrations of 60 μg/L for piroxicam and 50 μg/L for celecoxib) were applied in the synthetic samples. After determining the optimum conditions, the PIRO degradation efficiency under optimum conditions investigated to confirm the process's efficiency. The results indicated that for PIRO and CELE in synthetic samples the degradation rates were 96.08 % and 94.71 % respectively. Results shown that the degradation rate of piroxicam and celecoxib in the real wastewater sample were 89.6 % and 84.25 %, respectively. Therefore, electron beam irradiation is a long-lasting and promising method for removal emerging contaminants from wastewater, like non-steroidal anti-inflammatory drugs, that can't be removed by conventional wastewater treatment methods. Due to the economic limitations, the EB irradiation can be used in combination with conventional wastewater treatment methods to achieve the degradation rate along with the economic efficiency.

## CRediT authorship contribution statement

**Niloufar Borhani Yazdi:** Writing – review & editing, Writing – original draft, Investigation, Formal analysis, Data curation, Conceptualization. **Mohammad Rezvani Ghalhari:** Writing – review & editing, Writing – original draft, Visualization, Validation, Software, Formal analysis, Conceptualization. **Ali Parach:** Visualization, Validation, Resources, Methodology, Formal analysis. **Mohammad Hassan Ehrampoush:** Writing – original draft, Validation, Methodology, Formal analysis, Data curation, Conceptualization. **Kamal Ghadiri:** Writing – review & editing, Writing – original draft, Visualization, Validation, Resources, Methodology, Formal analysis, Conceptualization. **Mahdi Ghorbanian:** Writing – original draft, Resources, Methodology, Formal analysis, Data curation, Conceptualization. **Mohammad Hossein Zare Hassanabadi:** Writing – original draft, Validation, Resources, Conceptualization. **Ehsan Abouee Mehrizi:** Writing – review & editing, Writing – original draft, Visualization, Validation, Supervision, Software, Resources, Project administration, Methodology, Funding acquisition, Conceptualization.

## Consent to participate

Not applicable.

## Consent to publish

Not applicable.

## Availability of data and materials

All data generated or analyzed during this study are included in this published article.

## Funding

Research reported in this publication was supported by Elite Researcher Grant Committee under award number [9428] from 10.13039/501100015034Shahid Sadoughi University of Medical Sciences.

## Declaration of competing interest

The authors declare that they have no known competing financial interests or personal relationships that could have appeared to influence the work reported in this paper.
